# Foot health and quality of life among university students: cross-sectional study

**DOI:** 10.1590/1516-3180.2017.0264230917

**Published:** 2018-03-23

**Authors:** David Rodríguez-Sanz, Daniel Barbeito-Fernández, Marta Elena Losa-Iglesias, Jesús Luis Saleta-Canosa, Daniel López-López, Natalia Tovaruela-Carrión, Ricardo Becerro-de-Bengoa-Vallejo

**Affiliations:** I BSc, PT, MSc, PhD. Assistant Professor, Research Group, School of Health, Exercise and Sport, European University of Madrid, Villaviciosa de Odón, Madrid, Spain.; II BSc. External Collaborator and Research, Health and Podiatry Unit, Department of Health Sciences, School of Nursing and Podiatry, University of Coruña, Ferrol, Spain.; III BSc, RN, BSc, MSc, PhD. Full Professor, Faculty of Health Sciences, King Juan Carlos University, Alcorcón, Spain.; IV MD, PhD. Associate Professor, Clinical Epidemiology Research Group, Department of Health Sciences, School of Nursing and Podiatry, University of Coruña, Ferrol, Spain.; V BSc, BSc, MSc, PhD. Assistant Professor and Research, Health and Podiatry Unit, Department of Health Sciences, School of Nursing and Podiatry, University of Coruña, Ferrol, Spain.; VI DP, MSc, PhD. Assistant Professor, Department of Podiatry, University of Seville, Seville, Spain.; VII RN, BSc, MLIS, DPM, PhD, DHL. Full Professor, Department of Physiotherapy and Podiatry, School of Nursing, Physiotherapy and Podiatry, Complutense University of Madrid, Madrid, Spain.

**Keywords:** Foot, Primary health care, Quality of life

## Abstract

**BACKGROUND:**

Foot problems are believed to reduce quality of life and are increasingly present. Even among young adults of university age, untreated foot problems can lead to postural and mobility problems. Accordingly, our aim here was to determine the relationship between foot health and quality of life and general health among male and female university students.

**DESIGN AND SETTING:**

Observational cross-sectional quantitative study conducted at the Podiatric Medicine and Surgery Clinic of the University of Coruña, Ferrol, Spain.

**METHODS:**

A sample of 112 participants of median age 22 years came to a health center, where self-reported data were registered, including professional activity, and scores obtained through the Foot Health Status Questionnaire (FHSQ) were compared.

**RESULTS:**

In Section One of the FHSQ, the university students recorded lower scores of 66.66 in the footwear domain and 60 in the general foot health domain and higher scores of 84.37 in the foot pain domain and 93.75 in the foot function domain. In Section Two, they obtained lower scores of 60 in the overall health domain and 62.50 in the vigor domain and higher scores of 100 in the physical activity and 87.50 in the social capacity domain. Differences between males and females were evaluated using the Wilcoxon rank-sum test, which showing statistical significance (P < 0.05) regarding the dimensions of footwear and general foot health.

**CONCLUSIONS:**

These university students’ quality of life relating to foot health was poor. This appears to be associated with the university period, regardless of gender.

## INTRODUCTION

One of the most critical periods in human development is between the ages of 18 and 33 years, which is the bridge between childhood and adulthood.^1^ During this period of physical, psychological, social and sexual development, young people gradually assume responsibility for their own health.^2^ In addition, this age group has specific foot health issues that differ from those of other age groups, such as ankle sprains, tinea pedis, onychomycosis, plantar warts and ingrown toenails.^3^^,^^4^^,^^5^^,^^6^ They are also subjected to different kinds of general changes, such as greater autonomy, control over their lifestyle, control over physical activity and development of attitudes and beliefs about health and financial problems.^7^^,^^8^

Even at this age, untreated foot problems can lead to scoliosis, postural problems, slower walking speeds, uneven plantar pressure distribution, difficulty in carrying out daily activities, increased risk of falling and appearance of neurological diseases,^9^^,^^10^ all of which can affect these individuals’ academic achievement, quality of life, personal autonomy and wellbeing.^11^ Poor foot health is now recognized by the governments in general as an important public health issue because of its negative impact on individuals and on society. This includes difficulty in putting on shoes, pain, gait disorders, reduced walking speed, variation in plantar pressures and risk of falling.^12^^,^^13^^,^^14^

Despite the extent of this problem, the relationship between foot health and quality of life during this developmental period has not been studied. In the general population, the prevalence of foot health problems is between 71% and 87%. The problems relate to claw toes, hallux valgus, hammer toes, overlapping toes, hallux extensus, pes planus, Morton’s neuroma, tailor’s bunions, plantar fasciitis and pes cavus.^15^^,^^16^ Although these problems are multifactorial, they may predict loss of independence, vulnerability,^17^ defenselessness^18^ and reduced quality of life and wellbeing.

Based on these issues, it is important to consider illnesses and deformities of the foot, postural alterations and other underlying diseases as factors to be taken into account when planning treatment and preventive care activities. Moreover, there is a need for care and follow-up regarding foot health for university students that so far remains unattended. Such issues need to be addressed in seeking to ensure better quality of life and wellbeing for university students.

Thus, we sought to determine the state of foot health among male and female university students and its relationship to their overall quality of life and general health. At present little is known about the factors that affect the development of foot health. Foot problems are predisposing factors for the appearance of injuries in later life that could be prevented through implementing programs to improve the general condition of university students’ feet.

## METHODS

This study was approved by the Research Ethics Committee of the University of Coruña, in Coruña, Spain. All participants gave written informed consent. Ethical standards for research on human beings based on the Declaration of Helsinki (World Medical Association) and the Convention of the Council of Europe on human rights and biomedicine, as well as those based on UNESCO’s Universal Declaration on the Human Genome and Human Rights and those of other appropriate national or institutional organizations were adhered to.

### Respondents

Students between the ages of 18 and 33 years of similar socioeconomic level participated in this cross-sectional study between September 2014 and May 2015. Participants were recruited from the Clinic of Podiatric Medicine and Surgery (CPMS), which provides treatment for foot diseases and disorders at the University of Coruña, in Ferrol, Spain. Advertisements were placed on the CPMS website and in University newsletters. Information leaflets were provided to students clinicians, students from another health science college of the University like nursing, medicine or physiotherapy. Additionally, notices were placed on local bulletin boards.

These students came to the Podiatric Medicine and Surgery Clinic. They were eligible for inclusion in the study through a non-probability consecutive sampling technique. The inclusion criteria were that they needed to be healthy volunteers without any relevant medical records or family history and that they gave consent for enrollment into the study. The exclusion criteria included immunodepression, histories of trauma and foot surgery, neurological alterations and lack of or only partial autonomy in performing daily activities.^19^

### Data collection

All measurements were made by a single researcher. Height, weight and body mass index (BMI) were determined during the visit to the clinic. The students then completed the Foot Health Status Questionnaire (FHSQ). This self-administered questionnaire on health-related quality of life is intended specifically for the foot and has been recognized as a validated test.^20^^,^^21^ Foot-specific and general health-related quality of life was assessed using version 1.03 of the FHSQ,^22^ which contains three sections. The first section consists of 13 questions reflecting four domains relating to foot health (Appendix 1): foot pain, foot function, footwear and general foot health. The first section has demonstrated high degrees of content, criterion and construct validity (Cronbach α = 0.89-0.95) and high retest reliability (intraclass correlation coefficient = 0.74-0.92).^23^ It has been shown to be the most appropriate measurement of foot health-related quality of life for pathological skin and nail conditions and for neurological, orthopedic and musculoskeletal disorders, among others.^24^^,^^25^

Each domain has a specific number of questions (Appendix 2): four on pain, four on function, three on footwear and two on general foot health. The assessment of pain and function is based on physical phenomena; the evaluation of footwear uses practical aspects of availability and shoe comfort; and the perception of general foot health is based on the patients’ self-assessment of the state of their feet. Each question allows several answers, and these are placed on a Likert-type ordinal scale (words or phrases corresponding to a numerical scale). The descriptors for these scales vary for each domain, and the person completing the questionnaire should choose only one response, i.e. whichever response is thought to be the most appropriate. The questionnaire does not provide an overall score but, rather, it generates an index for each domain. To obtain these indices, the responses are analyzed through computer software (FHSQ, version 1.03). After processing the data, the software produces a score ranging from 0 to 100. A score of zero represents the worst state of health for the foot, while 100 is the best possible condition.

The second section of the FHSQ includes questions that reflect four general health-related domains (Appendix 2): general health, physical activity, social capacity and vigor. The domains and questions in this section are largely adapted from the Medical Outcomes Study 36-Item Short-Form Health Survey,^24^ which has been validated for use in the Australian population.^25^ Specific questions of the FHSQ that assess section 2 domains are shown in Appendix 2.

Lastly, the third section contains questions asking for sociodemographic data such as the participants’ age and sex and about their medical records.

### Sample size

The smallest clinically important difference in FHSQ scores is 21 points.^21^^,^^22^ Assuming a standard deviation of around 29 for a bilateral hypothesis and an alpha of 5%, at least 94 students would be needed to detect a 21-point difference with 80% power. Students were enrolled consecutively until the sample size was achieved.

### Statistical methods

Continuous variables were expressed as the median and interquartile range. Differences between men and women were compared using independent t tests if the data were normally distributed or the Wilcoxon rank-sum test if not. The data were analyzed using the SPSS statistics package. Alpha was set at 0.05, and all tests were two-tailed. The Foot Health Status Questionnaire version 1.03 was used to obtain quality-of-life scores relating to foot health.

## RESULTS

A total of 112 university students of less than 33 years of age were enrolled. The sample analyzed included 85 women (75.9%) and 27 men (24.1%) between 18 and 33 years of age. Most students were normal weight (BMI of 22.27 kg/m^2^). These results are shown in Table 1.

**Table 1:** Sociodemographic and clinical characteristics of the sample population

	Total group Median (IQ range) n = 112	Male Median (IQ range) n = 27	Female Median (IQ range) n = 85	P-value Male versus female
Age, years	22 (3)	23 (5)	22 (3)	0.562
Weight (kg)	63 (19.5)	80 (18)	58 (11)	0.000
Height (cm)	168 (12.5)	180 (7)	165 (10)	0.000
Body mass index (kg/m^2^)	22.27 (4.25)	24.03 (4.79)	21.80 (4.07)	0.009

IQ = interquartile.

Table 1 also shows the clinical domains of the FHSQ questionnaire and the sociodemographic characteristics of the informants. As can be seen, most of the informants were normal weight (BMI = 22.27 kg/m^2^). All variables showed non-normal distribution (P < 0.05), and therefore the Wilcoxon rank-sum test was used.

Table 2 shows a comparison of the scores obtained through the FHSQ. Section One of the FHSQ evaluated four specific foot domains, namely pain, function, health and footwear. The median scores were higher in relation to assessment of pain and function, and lower in relation to foot health and footwear. Section Two gave an assessment of four domains of general wellbeing: overall health, physical function, social capacity and vigor. In this section, the median foot pain scores for men and women were 87.50 and 81.25 respectively and the function scores for men and women were both 93.75, The foot health scores were 85 and 60 for men and women respectively, and the footwear scores were 75 and 58.33 for men and women respectively (Table 2). The median scores for physical function and social capacity were significantly lower than those for overall health and vigor, for both men and women. The differences between males and females were statistically significant (P < 0.05) for the dimensions of the FHSQ questionnaire that assessed footwear and general foot health. These results appear in Table 2.

**Table 2:** Comparisons of Foot Health Status Questionnaire survey scores for total group and gender groups

	Total group Median (IQ range) n = 112	Male Median (IQ range) n = 27	Female Median (IQ range) n = 85	P-value Male versus female
Foot pain	84.37 (18.12)	87.50 (15)	81.25 (24.37)	0.122
Foot function	93.75 (18.75)	93.75 (12.50)	93.75 (18.75)	0.127
Footwear	66.66 (41.66)	75 (18.333)	58.33 (41.66)	0.005
General foot health	60 (27.5)	85 (25)	60 (30)	0.037
Overall health	60 (20)	60 (20)	60 (20)	0.730
Physical activity	100 (5.56)	100 (5.56)	100 (5.56)	0.170
Social capacity	87.50 (25)	87.50 (25)	87.50 (25)	0.176
Vigor	62.50 (25)	68.75 (25)	62.50 (25)	0.103

IQ = interquartile.

## DISCUSSION

The purpose of this study was to determine the relationship between quality of life and foot health among male and female university students, given that the high prevalence of foot problems has been recognized by the governments as a threat to public health.

Foot health is essential to university students, in that it enables them to have greater autonomy, have control over their lifestyles and do physical activity.^26^ In a study on the population in Spain aged 40 years or older, the following prevalences of podiatric medical abnormalities were found: claw toe (69.7%), hallux valgus (38.0%) and hallux extensus (15.8%).^15^ The prevalences increased with age and were higher among females.^27^

The results from the few studies on this topic indicate that the impact of foot health on quality of life is not as obvious as it appears to be.^15^^,^^28^ On the other hand, our results confirm that female university students have lower median footwear and general health scores than those of men, thus indicating that they have poorer quality of life in relation to these two domains. We did not find any differences in any other domain. This finding is consistent with studies from other authors.^29^^,^^30^^,^^31^^,^^32^

Furthermore, our students had lower median scores for general health and vigor. This situation is associated with greater limitations in carrying out a wide range of physical activities, a lack of energy for participating in activities and an increased risk of becoming socially isolated.^8^^,^^22^

Given these results, it seems necessary to point out the importance of medical and podiatric care and follow-up, with the aim of preventing the appearance of illnesses and deformities of the foot. This is fundamental for enabling improvement of university students’ health, quality of life and autonomy.

We were unable to compare our results with those of other studies, given the differences in criteria and methodological variations, because we did not find any similar studies on quality of life relating to foot health.

This shows that there is a need for further research on this topic, in order to find out about the different therapeutic interventions used by professionals within podiatry and medicine that might improve foot health and quality of life, not only among university students but also in the general population.

Our study includes several important limitations that need to be acknowledged. Firstly, this study was performed in a clinic of podiatric medicine and surgery with a small number of participants. Secondly, expanding data collection to other countries may help to identify whether there is any culture in which this association does not exist and identify the mechanisms involved in foot health and health in general. Lastly, the recruitment methodology showed several drawbacks relating to the relatively small sample size. A more diverse sample, including individuals from several countries, would be beneficial for improving the strength of such studies.

This highlights the need for further research on the importance of medical and podiatric care and follow-up for the feet and for health in general, in order to prevent the appearance of illnesses and deformities of the feet and maintain the overall health of the body. This is fundamental for enabling improvement of university students’ health, quality of life, wellbeing and autonomy.

## CONCLUSIONS

Over this study period, these university students had poor quality of life relating to foot health. This appears to be associated with the university period, regardless of gender. Therefore, there is a need to develop foot health behavior that enables proper care and follow-up for the feet. This is extremely important for preventing the appearance or development of lesions, pain, infections or deformities, in order to enhance the quality of life of university students. Future studies seeking to identify significant factors influencing the quality of life relating to foot health among university students need to be pursued.
